# Oxygen carrying capacity of salvaged blood in patients undergoing off-pump coronary artery bypass grafting surgery: a prospective observational study

**DOI:** 10.1186/s13019-015-0330-x

**Published:** 2015-10-14

**Authors:** Xiu Liang Li, Peng Dong, Ming Tian, Jia Xiang Ni, Fang Gao Smith

**Affiliations:** 1 Department of Pain Management, Xuanwu Hospital of Capital Medical University, No. 45, Changchun Street, Xicheng District, 100053 Beijing, China; 2 Department of Anaesthesiology, Beijing Friendship Hospital, Capital Medical University, No. 95, Yong’an Road, Xicheng District, 100050 Beijing, China; 3 Department of Anaesthesiology, the 2nd Affiliated Hospital & Yuying Children Hospital of Wenzhou Medical University, Wenzhou, China; 4 Perioperative, Critical Care and Trauma Trials Group, University of Birmingham, Edgbaston B15 2WB, Birmingham, UK

**Keywords:** Intraoperative cell salvage, Oxyhemoglobin affinity, 2,3-disphosphoglycerate, Free hemoglobin

## Abstract

**Background:**

Intraoperative cell salvage (ICS), hereby referred to ‘mechanical red cell salvage’, has been widely used and proven to be an effective way to reduce or avoid the need for allogeneic red blood cells (RBCs)transfusion and its associated complications in surgeries involving major blood loss. However, little is known about the influence of this technique on the functional state of salvaged RBCs. Furthermore, there are no articles that describe the change of free hemoglobin (fHb) in salvage blood during storage, which is a key index of the quality control of salvaged blood. Therefore, in this study, the influence of ICS on the function of salvaged RBCs and the changes of salvaged RBCs during storage were studied with respect to the presence of oxyhemoglobin affinity (recorded as a P_50_ value) and the level of 2, 3-diphosphoglycerate (2, 3-DPG) and fHb by comparing salvaged RBCs with self-venous RBCs and 2-week-old packed RBCs.

**Methods:**

Fifteen patients undergoing off-pump coronary artery bypass grafting (OPCAB) surgery were enrolled. Blood was collected and processed using a Dideco Electa device. The level of P_50_, 2, 3-DPG and fHB from salvaged RBCs, venous RBCs and 2-week-old packed RBCs was measured. We also measured the changes of these indicators among salvaged RBCs at 4 h (storage at 21–24 °C) and at 24 h (storage at 1–6 °C).

**Results:**

The P_50_ value of salvaged RBCs at 0 h (28.77 ± 0.27 mmHg) was significantly higher than the value of venous RBCs (27.07 ± 0.23 mmHg, *p =* 0.000) and the value of the 2-week-old packed RBCs (16.26 ± 0.62 mmHg, *p =* 0.000). P_50_ value did not change obviously at 4 h (*p =* 0.121) and 24 h (*p =* 0.384) compared with the value at 0 h. The 2, 3-DPG value of salvaged RBCs at 0 h (17.94 ± 6.91 μmol/g Hb) was significantly higher than the value of venous RBCs (12.73 ± 6.52 mmHg, *p =* 0.007) and the value of the 2-week-old packed RBCs (2.62 ± 3.13 mmHg, *p =* 0.000). The level of 2, 3-DPG slightly decreased at 4 h (*p =* 0.380) and 24 h (*p =* 0.425) compared with the value at 0 h. Percentage of hemolysis of the salvaged blood at 0 h(0.51 ± 0.27 %) was significantly higher than the level of venous blood (0.07 ± 0.05 %, *p =* 0.000) and the value of 2-week-old packed RBCs (0.07 ± 0.05 %, *p =* 0.000), and reached 1.11 ± 0.42 % at 4 h (*p =* 0.002) and 1.83 ± 0.77 % at 24 h (*p =* 0.000).

**Conclusions:**

The oxygen transport function of salvaged RBCs at 0 h was not influenced by the cell salvage process and was better than that of the venous RBCs and 2-week-old packed RBCs. At the end of storage, the oxygen transport function of salvaged RBCs did not change obviously, but percentage of hemolysis significantly increased.

## Background

Currently, increasing recognition of the morbidity and mortality associated with allogeneic RBCs transfusion in patients undergoing coronary artery bypass grafting (CABG) surgery has gained increasing attention in clinical setting [1–3]. Moreover, shortages of allogeneic RBCs have become a challenge for surgeries involving major blood loss. ICS as a blood conservation technique has been widely used and proven to be an effective way to reduce or avoid the need for allogeneic RBCs transfusion and its associated complications in cardiac surgery, orthopedic surgery, liver transplantation and neurosurgery [4–8]. However, little is known about the influence of this cell-saving technique on the functional state of salvaged RBCs, especially on the oxygen carrying capacity and their contents of 2, 3-DPG. Falling levels of red cell 2, 3-DPG resulting in the increase in oxygen affinity and the left shift of hemoglobin oxygen dissociation curve (HODC) can influence the oxygen from RBCs to body tissues [9, 10]. Furthermore, the level of fHb, as a measure of hemolysis, is a key index of the quality control of stored RBCs, and associated with renal dysfunction and an increased risk of death [11–14]. There are no articles that describe the effect of storage time on the level of fHb in salvage blood (at room temperature 21–24 °C for up to 4 h and at 1–6 °C for up to 24 h) [15].

Therefore, firstly, the aim of the present study was to examine how in vitro the cell salvage procedure influences P_50_ value as well as the 2, 3-DPG contents by comparing salvaged RBCs with self-venous RBCs and 2-week-old packed RBCs. Secondly, this study also was aimed to observe the changes of fHb, P_50_ and 2, 3-DPG during storage; the results from our study may provide a basis for the safe use of salvaged RBCs in the clinical setting.

## Methods

### Study participants

This study was approved by the local Institutional Review Board of the Beijing Friendship Hospital. After written informed consent was obtained, 15 Patients scheduled for OPCAB surgery were enrolled. An OPCAB surgery is a procedure that does not require the use of the cardiopulmonary bypass (CPB) machine [16]. Exclusion criteria were patients less than 18 years or over 80 years old, emergency operation, hematopathy, the use of CPB, preoperative and intraoperative allogeneic RBCs transfusion. All patients were operated by one group surgeons. We used the Dideco Electa autotransfusion device in all patients because the function of washed erythrocytes differs widely from one device to another [17].

### Cell salvage procedures

The Dideco Electa device (SN: BO15214H05, 42037 Mirandola Italy) was set up and run according to the manufacturer’s instructions. We selected the automatic mode and used a BT225-mL bowl operated with a centrifuge speed of 5600 rpm. The bowl filled at a speed of 400 ml/min, washed at rate of 500 ml/min with a wash volume of 1000 ml and emptied at a speed 400 ml/min. The fill, wash and empty rates were strictly followed to maintain consistency between samples. The blood was heparinized with 0.9 % saline (30 000 IU of heparin in 1000 mL of 0.9 % saline) and washed also with 0.9 % saline [18, 19]. Before blood collection, vacuum suction was limited to −0.2 bar to prevent excessive hemolysis, the suction line and the reservoir were rinsed with 150 ml of 0.9 % heparinized saline and during collection the heparinized solutions were added to the blood in a ratio of approximately 1:7. One trained executive operator accomplished all the blood salvage process.

### Blood sample collection

All blood samples were taken, respectively, from the central venous line after induction of anesthesia, from the reinfusion bag at 0 h, 4 h (storage at 21–24 °C) and 24 h (storage at 1–6 °C) for the measurement of P_50_, 2, 3-DPG, fHb and blood gas analysis. Blood samples were also drawn from 2-week-old packed RBCs which were supplied by blood bank of Beijing Friendship Hospital and had been stored for 2 weeks at 1–6 °C. Packed RBCs were prepared by whole bloods which were centrifuged for 7 min with a centrifugal force of 5000 × g at 4 ± 2 °C, and were added with acid-citrate-dextrose solution (citrate 4.8 g/L, sodium citrate 13.2 g/L and dextrose 14.7 g/L). Packed RBCs were not leukocyte-reduced and washed. All blood samples were analyzed within 20 min after collection by one person.

### Laboratory measurements

#### Free hemoglobin

The fHb was measured with the HemoCue Plasma Low/Hb Photometer (HemoCue, Inc., Angelholm, Sweden) which is a sensitive test method and can detect down to 0 · 0 g/dl [11]. Hb concentrations were measured using a HemoCue Hb 201 + Analyzer (Kuvettgatan1, Angelholm, Sweden). The percentage of hemolysis was calculated using the following formula: Percentage of hemolysis (%) = [(1-Hct) × fHb/Hb] × 100 % [20].

#### Determination of P_50_

Hemoglobin Oxygen dissociation curve were performed by the method of Opdahl H [21]. In brief, the blood samples were divided into two 5 mL plastic syringes (2.0 mL each) which were equilibrated with a gas containing 5 % CO _2_ and 20 % O _2_ in nitrogen or 5 % CO _2_ in nitrogen, respectively, for 20 min. The samples of the two syringes were then transferred anaerobically into 2 mL syringes containing a glass bead for blood gas analysis. The measured PO_2_ was corrected for standard conditions (pH 7.4, temperature 37 °C, PaCO_2_40mmHg) according to the equation: △logPO_2_ = −0.48△pH + 0.0013BE + 0.024△T [22]. HODC was drawn from five to seven sets of corresponding SO _2_ - PO _2_ values.

The sigmoid HbO_2_ dissociation curve was converted to a rectilinear one according to the Hill equation which is approximately valid in the range 30 to 70 % SO2: log [Y/(100-Y)] = log K+ n log P, where Y is oxygen saturation, K is the equilibrium constant for the combination of O _2_ with Hb, n is an expression of “heme-heme interaction” and P is the Po_2_ value at which Y is measured [23, 24]. When the data are plotted in this way, a straight line is obtained; n is determined by the slope of the line; P_50_ which is the PO_2_ corresponding to half saturation of the Hb with oxygen is obtained from the intercept of the line at the abscissa log [Y/(100-Y)] =0 [10].

The 2, 3 DPG was measured by Ultraviolet test according to the manufacturer’s instructions (Roche Diagnostics GmbH, Mannheim, Germany). Plasma sodium, potassium, calcium, lactate and Chloride concentrations were assayed in the GEM Premier 3000 Blood Gas/Electrolyte Analyzer (Instrumentation Laboratory Company, 101 Hartwell Avenue Lexington, Massachusetts, USA).

### Statistical analysis

Data were analyzed using SPSS15.0 for Windows (SPSS, Inc., Chicago, USA) and presented as mean ± SD. The differences between venous RBCs and salvaged RBCs were analyzed using paired-samples *T* test. The differences between 2-week-old packed RBCs and venous RBCs, salvaged RBCs were analyzed using independent-samples *T* test. The changes during the storage of salvaged RBCs were analyzed using one-way analysis of variance (ANOVA). When ANOVA indicated that effects were significant, post hoc analysis was performed using the LSD approach. *P <*0.05 was considered statistically significant.

## Results

From August 2013 to February 2014, fifteen eligible patients were enrolled in this study. They were men and the mean age was 62.3 ± 8.5 (ranging from 51 to 76 years).

### The change of P_50_ level and 2, 3-DPG level

The change of P_50_ level and 2, 3-DPG level was presented in Table 1.

**Table 1:** P_50_ level and 2, 3-DPG level

	P_50_ (mmHg)	2, 3-DPG (μmol/g Hb)
salvaged RBCs 0 h	27.07 ± 0.23	12.73 ± 6.52
salvaged RBCs 4 h	28.77 ± 0.27	17.94 ± 6.91
salvaged RBCs 24 h	28.94 ± 0.25	15.17 ± 11.61
venous RBCs	28.87 ± 0.34	15.38 ± 5.85
2-week-old packed RBCs	16.26 ± 0.62	2.62 ± 3.13

The P_50_ level of venous RBCs and salvaged RBCs 0 h, 4 h, 24 h and 2-week-old packed RBCs was, respectively, 27.07 ± 0.23, 28.77 ± 0.27, 28.94 ± 0.25, 28.87 ± 0.34 and 16.26 ± 0.62 mmHg. There was thus general increase in P_50_ level at salvaged RBCs, the increase at 0 h, 4 h and 24 h was significant compared with the P_50_ level at venous RBCs (*p =* 0.000, respectively), and the increase at 0 h, 4 h and 24 h was also significant compared with the P_50_ level at 2-week-old packed RBCs (*p =* 0.000, respectively). P_50_ value did not change obviously at 4 h (*p =* 0.121) and 24 h (*p =* 0.384) compared with the value at 0 h.

The 2, 3-DPG level of venous RBCs and salvaged RBCs 0 h, 4 h, 24 h and 2-week-old packed RBCs was, respectively, 12.73 ± 6.52, 17.94 ± 6.91, 15.17 ± 11.61, 15.38 ± 5.85 and 2.62 ± 3.13 μmol/g Hb. There was thus general increase in 2, 3-DPG level at salvaged RBCs, the increase was significant at 0 h (*p =* 0.007), and not significant at 4 h (*p =* 0.272) and at 24 h (*p =* 0.099)compared with the value of venous RBCs, and the increase at 0 h, 4 h and 24 h was significant compared with the 2, 3-DPG level at 2-week-old packed RBCs (*p =* 0.000, respectively). The level of 2, 3-DPG slightly decreased at 4 h (*p =* 0.380) and 24 h (*p =* 0.425) compared with the value at 0 h.

### The changes of salvaged RBCs during storage

The changes of salvaged RBCs during storage were presented in Table 2.

**Table 2:** Concentration of fHb, percentage of hemolysis, the value of potassium and pH value

	fHB (g/L)	Percentage of hemolysis (%)	Potassium (mmol/L)	pH
salvaged RBCs 0 h	1.04 ± 0.52	0.51 ± 0.27	0.59 ± 0.26	7.51 ± 0.07
salvaged RBCs 4 h	2.10 ± 0.74	1.11 ± 0.42	1.69 ± 0.38	7.43 ± 0.05
salvaged RBCs 24 h	3.69 ± 1.18	1.83 ± 0.77	7.18 ± 2.53	7.42 ± 0.08
venous RBCs	0.12 ± 0.09	0.07 ± 0.05	4.34 ± 0.37	7.33 ± 0.06
2-week-old packed RBCs	0.19 ± 0.13	0.07 ± 0.05	16.56 ± 3.58	6.50 ± 0.06

The mean fHB level was 0.12 ± 0.09 g/L in venous RBCs, 0.19 ± 0.13 g/L in 2-week-old packed RBCs, 1.04 ± 0.52 g/L in 0 h salvaged RBCs and increased throughout storage time, reached 2.10 ± 0.74 g/L at 4 h (compared with the value at 0 h, *p =* 0.003), 3.69 ± 1.18 g/L at 24 h (compared with the value at 0 h, *p =* 0.000).

The percentage of hemolysis of salvaged RBCs at 0 h was 0.51 ± 0.27 %, and was significantly higher than the value of venous RBCs (0.07 ± 0.05 %, *p =* 0.000) and the value of 2-week-old packed RBCs (0.07 ± 0.05 %, *p =* 0.000), and increased throughout storage time, reached 1.11 ± 0.42 % at 4 h (compared with the value at 0 h, *p =* 0.002), 1.83 ± 0.77 % at 24 h (compared with the value at 0 h, *p =* 0.000).

The potassium level was 0.59 ± 0.26 mmol/L in 0 h salvaged RBCs and increased throughout storage time, reached 1.69 ± 0.38 mmol/L at 4 h (compared with the value at 0 h, *p =* 0.000), 7.18 ± 2.53 mmol/L at 24 h (compared with the value at 0 h, *p =* 0.000). But the potassium level at 4 h and 24 h was still lower compared with the value of 2-week-old packed RBCs (*p =* 0.000, respectively).

The pH level in 0 h salvaged RBCs was 7.51 ± 0.07, decreased throughout storage time and reached 7.43 ± 0.05 at 4 h (compared with the value at 0 h, *p =* 0.015) and 7.42 ± 0.08 at 24 h (compared with the value at 0 h, *p =* 0.014). The pH level at 4 h and 24 h was still higher than the value of 2-week-old packed RBCs (*p =* 0.000, respectively).

## Discussion

ICS has become an important autotransfusion method in cardiac surgery and the safe issue also has been extensively studied [4, 5, 25, 26]. However, little attention has been paid to the oxygen transport function of salvaged RBCs which should be the highest priority during salvage. In this preliminary work, we studied the P_50_ and 2, 3-DPG level from salvaged RBCs and venous RBCs via a self-control study to determine the influence of the cell salvage process on salvaged RBCs. P_50_ is widely used as a criterion for assessing the binding affinity of hemoglobin for O_2_ and 2, 3-DPG is a key substance in regulating the oxygen delivery in vivo [17]. The results showed that the P_50_ value of salvaged RBCs at 0 h significantly increased compared with the value of venous RBCs and 2-week-old packed RBCs, and the 2, 3-DPG value also significantly increased compared with the value of venous RBCs and 2-week-old packed RBCs. Thus, the oxygen transport function of salvaged RBCs at 0 h was not influenced by the cell salvage process and was better than that of venous RBCs and 2-week-old packed RBCs.

The reason why the P_50_ and 2, 3-DPG level increased in salvaged RBCs at 0 h probably was that the vast majority of salvaged RBCs were young RBCs and old RBCs were eliminated during the operative procedure and the cell salvage progress. Young RBCs have higher P_50_ and 2, 3-DPG level than old RBCs [9, 10]. Haidas reported that the P_50_ and 2, 3-DPG level in young RBCs, old RBCs and total venous RBCs were, respectively, 31.2, 25.1 and 29.3 mmHg, 17.2, 7.3 and 14.7 μmol/g Hb [9]. Bunn also reported that the level of 2, 3-DPG in young cells and old cells separated by centrifugation was 17.1 and 12.3 μmol/g Hb [10]. In our study, the P_50_ and 2, 3-DPG level in venous RBCs and 0 h salvaged RBCs were, respectively, 27.07 and 28.77 mmHg, 12.73 and 17.94 μmol/g Hb. These results aligned well with studies of Haidas and Bunn. Furthermore, Che et al. also reported that in salvaged RBCs at 0 h, the 2, 3-DPG level was higher than that of venous RBCs (4.88vs. 4.38 mmol/L, *P <* 0.05) [20]. However, there are some contrary reports [17, 27]. Gu et al. thought that the high shear stress generated during the cell saver procedure may result in the 2, 3-DPG depletion, from 9.2 μmol/g Hb in venous RBCs to 5.41 μmol/g Hb in the salvaged RBCs [27]. But, cardiopulmonary bypass (CPB) was routinely used in their study and allogeneic RBCs were transfused in 6 patients. CPB can decrease 2, 3-DPG content of RBCs and in our study, the 2, 3-DPG level in 2-week-old packed RBCs had dropped to 2.62 ± 3.13 μmol/g Hb (0.0 in 6 patients) [28, 29]. So, the use of CPB and allogeneic RBCs may influence the accuracy of their results and was excluded in our study.

2-week-old packed RBCs often develop storage lesions. Glucose in these RBCs is consumed, levels of DPG and ATP decrease, while potassium levels increase [10, 30, 31]. In our study, 2-week-old packed RBCs also had these changes. Compared with 2-week-old packed RBCs, salvaged RBCs at 0 h had better oxygen transport function. When 2, 3-DPG-poor RBCs are transfused, only 43.89 % of the final level of 2, 3-DPG is resynthesized within 6 h after the end of transfusion [32]. Liang also reported that salvaged RBCs had better morphologic results than 2-week-old packed RBCs [8]. Observational studies in trauma, critical illness and cardiac surgery, linking the administration of allogeneic RBCs to increased mortality, support the concern that transfusion of allogeneic RBCs may be detrimental [1–3, 33–35]. A meta analysis showed that the use of ICS reduced the rate of exposure to allogeneic RBC transfusion by a relative 38 % and resulted in an average saving of 0.68 units of allogeneic RBC per patient [36]. So in CABG surgery with probable hemorrhage, ICS should as much as possible be used in order to avoid the transfusion of 2, 3-DPG-poor allogeneic RBCs.

Measurement of fHb and percentage of hemolysis has been used as a criterion for the quality control. An official guideline for acceptable level of hemolysis for salvaged blood has not been established. However, the quality standard level of hemolysis rate has been referred to packed blood < 1 % in USA or < 0.8 % in Europe [37]. In this study, percentage of hemolysis was 0.51 ± 0.27 % in salvaged RBCs at 0 h, which was well below 1 % or 0.8 % level. The increase in the level of fHb could be injurious by several potential mechanisms. Firstly, fHb can react with NO about 1000 times faster than with intact erythrocytes in a near-diffusion limited reaction to inhibit NO signaling and even at concentrations below 10μmmol/L (in heme)still can produce potent vasoconstriction when infused into the rat circulation [38]. Secondly, when hemoglobin is released into the extracellular compartment, ferrous heme is oxidized to ferryl heme protoporphyrin radical species which are potent oxidants that cause lipid peroxidation. Furthermore, fHb affinity for O_2_ is higher than intracorpuscular Hb (P_50_:20.50 vs.26.72 mmHg, *P <* 0.05) [10]. In clinical studies, elevated fHb concentration was independently and significantly correlated with acute kidney injury during major aortic surgery and an increased risk of death in critically ill patients with sepsis [12–14].

In this study, we also studied the changes of salvaged RBCs during storage. P_50_ level at 4 h and 24 h did not change obviously compare to that at 0 h. fHB level significantly increased and reached 2.10 ± 0.74 g/L at 4 h and 3.69 ± 1.18 g/L at 24 h. The level of potassium also significantly increased at the end of storage. The significant increase in the level of fHB and potassium was mainly due to the increase in osmotic fragility of salvaged RBCs which resulted in the increase in the susceptibility of RBCs to lysis [39]. In previous studies, cell saver salvaged blood stored at room temperature for 6 h and at 1–6 °C for 24 h after collection was transfused into infants undergoing open heart surgery without any relevant complications [40, 41]. However, these high fHB levels may not be acceptable for patients with endothelial dysfunction. Yu et al. reported that fHB did not cause systemic hypertension in healthy wild-type mice but induced severe systemic vasoconstriction in mice with endothelial dysfunction [42, 43]. They thought that reduced vascular nitric oxide levels associated with endothelial dysfunction sensitized mice to the vasoconstrictor effects of fHb administration [43].

Our study had limitations; firstly, we only measured effects of the Dideco Electa device on the quality of salvaged RBCs in patients undergoing OPCAB surgery, however the quality of washed RBCs is affected by the operative procedure and the type of device [17, 44]. Secondly, the study had a small sample size which may result in higher standard deviation of 2,3-DPG value. Further studies with larger sample size in different types of surgeries and devices are needed to confirm these outcomes and to provide a basis for the safe use of salvaged RBCs in the clinical setting.

## Conclusions

In summary, this study had shown that the oxygen transport function of salvaged RBCs at 0 h was not influenced by the cell salvage process and was better than that of the venous RBCs and 2-week-old packed RBCs. At the end of storage, the oxygen transport function of salvaged RBCs did not change obviously, but percentage of hemolysis significantly increased.
